# Curcuminoid WZ35 synergize with cisplatin by inducing ROS production and inhibiting TrxR1 activity in gastric cancer cells

**DOI:** 10.1186/s13046-019-1215-y

**Published:** 2019-05-21

**Authors:** Wei He, Yiqun Xia, Peihai Cao, Lin Hong, Tingting Zhang, Xin Shen, Peisen Zheng, Huanpei Shen, Guang Liang, Peng Zou

**Affiliations:** 1 Chemical Biology Research Center, School of Pharmaceutical Sciences, Wenzhou Medical University, Wenzhou, 325035 Zhejiang China; 2 Department of Digestive Diseases, The First Affiliated Hospital of Wenzhou Medical University, Wenzhou, 325035 Zhejiang China

**Keywords:** Thioredoxin reductase 1, Reactive oxygen species, WZ35, Cisplatin, Gastric cancer

## Abstract

**Background:**

Cisplatin is one of the most widely used chemotherapeutic agents, but its efficacy is limited by its side effects. Hence, it is of great significance to develop novel agents to synergize with cisplatin and decrease side effects. In our previous study, we demonstrated that WZ35, a novel curcumin analogue, exhibited potent anti-cancer effects in vitro and in vivo. Here, we investigated whether WZ35 synergize to potentiate cisplatin activity in gastric cancer cells.

**Methods:**

Cell apoptosis and cellular ROS levels were analyzed by flow cytometry. TrxR1 activity in gastric cells or tumor tissues was determined by the endpoint insulin reduction assay. Western blot was used to analyze the levels of indicated molecules. Nude mice xenograft model was used to test the effects of WZ35 and cisplatin combination on gastric cancer cell growth in vivo.

**Results:**

We found that WZ35 significantly enhanced cisplatin-induced cell growth inhibition and apoptosis in gastric cancer cells. Further mechanism study showed that WZ35 synergized the anti-tumor effects of cisplatin by inhibiting TrxR1 activity. By inhibiting TrxR1 activity, WZ35 combined with cisplatin markedly induced the production of ROS, activated p38 and JNK signaling pathways, and eventually induced apoptosis of gastric cancer cells. In vivo, WZ35 combined with cisplatin significantly suppressed tumor growth in a gastric cancer xenograft model, and effectively reduced the activity of TrxR1 in tumor tissues. Remarkably, WZ35 attenuated the body weight loss evoked by cisplatin treatment.

**Conclusion:**

This study elucidated the underlying mechanisms of synergistic effect of WZ35 and cisplatin, and suggest that such a combinational treatment might potentially become a more effective regimen in gastric cancer therapy.

**Electronic supplementary material:**

The online version of this article (10.1186/s13046-019-1215-y) contains supplementary material, which is available to authorized users.

## Background

Gastric cancer remains one of the leading cause of cancer-related deaths in the world [1, 2]. Surgery is the mainstay of treatment for localized gastric cancer, but recurrence rates are high even after radical resection [3]. Furthermore, most patients already present with local or distant metastases when first diagnosed with gastric cancer. Thus, adjuvant chemotherapy have been prescribed for gastric cancer and have shown considerable benefits in reducing cancer recurrence and increasing long-term survival [4, 5]. Cisplatin is a highly effective chemotherapeutic agent that is widely used in clinical therapeutic regimens for a variety of cancers, including gastric cancer. However, its application is limited by drug resistance and side effects, including genotoxicity, nephrotoxicity and acute myelotoxicity [6, 7]. Hence, it is of great significance to develop new agents to synergize with cisplatin and decrease side effects.

Cancer cells usually generate and maintain higher levels of ROS compared to normal cells. Elevated ROS levels render cancer cells more sensitive to agents that increase ROS generation [8, 9]. Therefore, stimulation of ROS is a potential therapeutic strategy for cancer, as exemplified by the approved anti-cancer drugs paclitaxel [10], sorafenib [11], and osimertinib [12]. Exploring the molecular mechanisms of ROS based treatment are required to improve the efficacy and specificity of anti-cancer drugs. WZ35, a novel curcumin analogue, has been reported to inhibit the proliferation of a variety of cancer cells in vitro and in vivo. Our previous study have suggested that ROS play a critical role in WZ35-induced apoptosis in cancer cells [13]. Until now, the molecular mechanism underlying WZ35-induced ROS production remains unclear.

The mammalian thioredoxin reductase 1 (TrxR1) is a selenocysteine (Sec) containing flavoenzyme, and has a critical role in regulating intracellular redox balance [14]. TrxR1 is upregulated in a number of human tumors, and may be associated with aggressive tumor growth and poor outcome [15, 16]. TrxR1 inactivation by chemical inhibition reverses tumor growth and sensitizes cancer cells to chemotherapeutic drugs [17–20]. Hence, TrxR1 has emerged as a attractive therapeutic target for anti-cancer drug development.

In the present study, we investigated whether WZ35 could synergize to enhance the anti-tumor effects of cisplatin in gastric cancer cells. We found that WZ35 combined with cisplatin exhibited potent synergistic effect to inhibit the proliferation of human gastric cancer cells, and further demonstrated that TrxR1 activity was involved in the synergistic effect in vitro and in vivo. Our study elucidated the underlying mechanisms of synergistic activity of WZ35 and cisplatin, and suggest that such a combinational treatment might potentially become a more effective regimen in gastric cancer therapy.

## Materials and methods

### Cell culture and reagents

Human gastric cancer cell lines SGC-7901 and BGC-823 were purchased from the Institute of Biochemistry and Cell Biology, Chinese Academy of Sciences. The cells were routinely cultured in RPMI 1640 medium containing 10% fetal bovine serum at 37 °C in a humidified incubator with an atmosphere of 5% CO_2_. N-acetyl-L-cysteine (NAC), L-glutathione (GSH) and cisplatin were purchased from Sigma (St. Louis, MO, USA). BMS-582949 and SP600125 were purchased from Selleck Chemicals (Houston, TX, USA). WZ35 was synthesized in our laboratory as previously described [13]. Antibodies including anti-Bcl-2 (sc-7382, 1:50), anti-TrxR1 (sc-28,321, 1:200), anti-GAPDH (sc-47,724, 1:200), mouse anti-rabbit IgG-HRP (sc-2357, 1:2000) and m-IgGκ BP-HRP (sc-516,102, 1:2000) were purchased from Santa Cruz Biotechnology (Santa Cruz, CA, USA). Antibodies including anti-p-p38 (4631, 1:1000), anti-p38 (9212, 1:1000), anti-p-JNK (4668, 1:1000) and anti-JNK (9252, 1:1000) were purchased from Cell Signaling Technology (Danvers, MA, USA). The anti-Ki-67 (ab15580, 1:1000) antibody was purchased from Abcam (Cambridge, MA, USA). FITC Annexin V apoptosis Detection Kit I and Propidium Iodide (PI) were purchased from BD Pharmingen (Franklin Lakes, NJ, USA).

### Cell viability assay

Cells were seeded into 96-well culture plates at a density of 8 × 10^3^ per well for 24 h. After indicated treatments, cell viability was examined by methyl thiazolyl tetrazolium (MTT) assay. The combination index (CI) of drug interaction was determined using the Chou-Talalay method [21]: CI = 1, additive interaction; CI > 1, antagonistic interaction; and CI < 1, synergistic interaction.

### Measurement of intracellular ROS

Cells were seeded into 6-well culture plates for 24 h, and then treated with WZ35, cisplatin, or the combination for 2 h. Cells were stained with 10 μM DCFH-DA (Beyotime Biotech, Nantong, China) for 30 min. Then the cells were collected and the fluorescence intensity was analyzed using a FACSCalibur flow cytometer (BD Biosciences, CA, USA). In some experiments, cells were pretreated with 5 mM NAC or GSH for 2 h prior exposure to compounds.

### Cell apoptosis analysis

Cells were seeded into 6-well culture plates for 24 h, and then treated with WZ35, cisplatin, or the combination for 24 h. Then the cells were harvested, washed twice with ice-cold phosphate-buffered saline (PBS), and evaluated for apoptosis by double staining with annexin V-FITC and propidium iodide in binding buffer for 30 min using a FACSCalibur flow cytometer.

### Western blot analysis

Cells were grown on 6-well culture plates and were treated with WZ35, cisplatin, or the combination for the indicated times. The cells were then washed once with 1 mL of PBS, and were lysed using cell lysis buffer. For western blot analysis, equal amounts of protein in each sample were separated by sodium dodecyl sulfate-polyacrylamide gel electrophoresis and electroblotted onto polyvinylidene difluoride membrane. The membranes were blocked using 5% nonfat milk at room temperature for 2 h and then incubated with primary antibodies at 4 °C overnight. Then, the membranes were washed three times with TBST, and incubated with the peroxidase-conjugated secondary antibodies for 1 h at room temperature. The immunoreactive bands were visualized using an ECL detection kit (Bio-Rad Laboratories, CA, USA).

### Docking of WZ35 to the TrxR1 structural model

To study the interaction between the WZ35 and TrxR1, a covalent dock was implement by CovalentDock [22]. The crystal structure of human TrxR1 (PDB code 2ZZ0, chain A) was used for present docking study. The center coordination of dock pocket was set as − 29.11, − 1.26, and − 6.55 which calculated by selecting residue Cys-497 and Cys-498. A grid box size of 60 × 60 × 60 points with a spacing of 0.375 Å between the grid points was implemented. The default parameters were used for running the docking simulation.

### Measurement of TrxR activity in cells or tumor tissues

TrxR activity in cell lysates or tumor tissues was measured by the endpoint insulin reduction assay as previously described [23]. The activity was expressed as the percentage of the control.

### In vivo antitumor study

Athymic nude mice (nu/nu, 5–6 week, female) were used for in vivo experiments. All animals were handled according to the Institutional Animal Care and Use Committee (IACUC) guidelines, Wenzhou Medical University. SGC-7901 cells (5 × 10^6^ cells in 100 μL of PBS) were harvested and injected subcutaneously into the right flank of mice. Mice were treated with WZ35, cisplatin, or the combination by intraperitoneal (i.p.) injection once every other day at the indicated doses. At the end of experiment, the mice were sacrificed and tumor tissues were excised and weighed. The tumor volume was determined by measuring length (l) and width (w) at the indicated time points.

### In vivo toxicity study

ICR mice (23–25 g, female, total *n* = 20) were used for in vivo toxicity study. All animals were handled according to the Institutional Animal Care and Use Committee (IACUC) guidelines, Wenzhou Medical University. The mice were divided into four experimental groups on randomization and blinding with five mice in each group. Mice were treated with WZ35, cisplatin, or the combination by intraperitoneal (i.p.) injection once every other day at the indicated doses. At the end of experiment, the mice were sacrificed and some organs were excised and weighed.

### Immunohistochemistry assay

The harvested tumor tissues were fixed in 4% paraformaldehyde for 24 h. Fixed tissues were embedded in paraffin and cut into 5-μm sections. Tissue sections were stained with the indicated antibodies. The signal was detected by biotinylated secondary antibodies, and colour was developed using DAB (3,3′-diaminobenzidine).

### Statistical analysis

All experiments were performed in triplicate. The data are reported as means ± SEM. All statistical analyses were performed using GraphPad Prism 5.0. Student’s t-test and two-way ANOVA were employed to analyze the differences between data sets. A *p* value < 0.05 was considered statistically significant.

## Results

### WZ35 synergistically augmented the cytotoxicity of cisplatin in gastric cancer cells

**Fig. 1:**
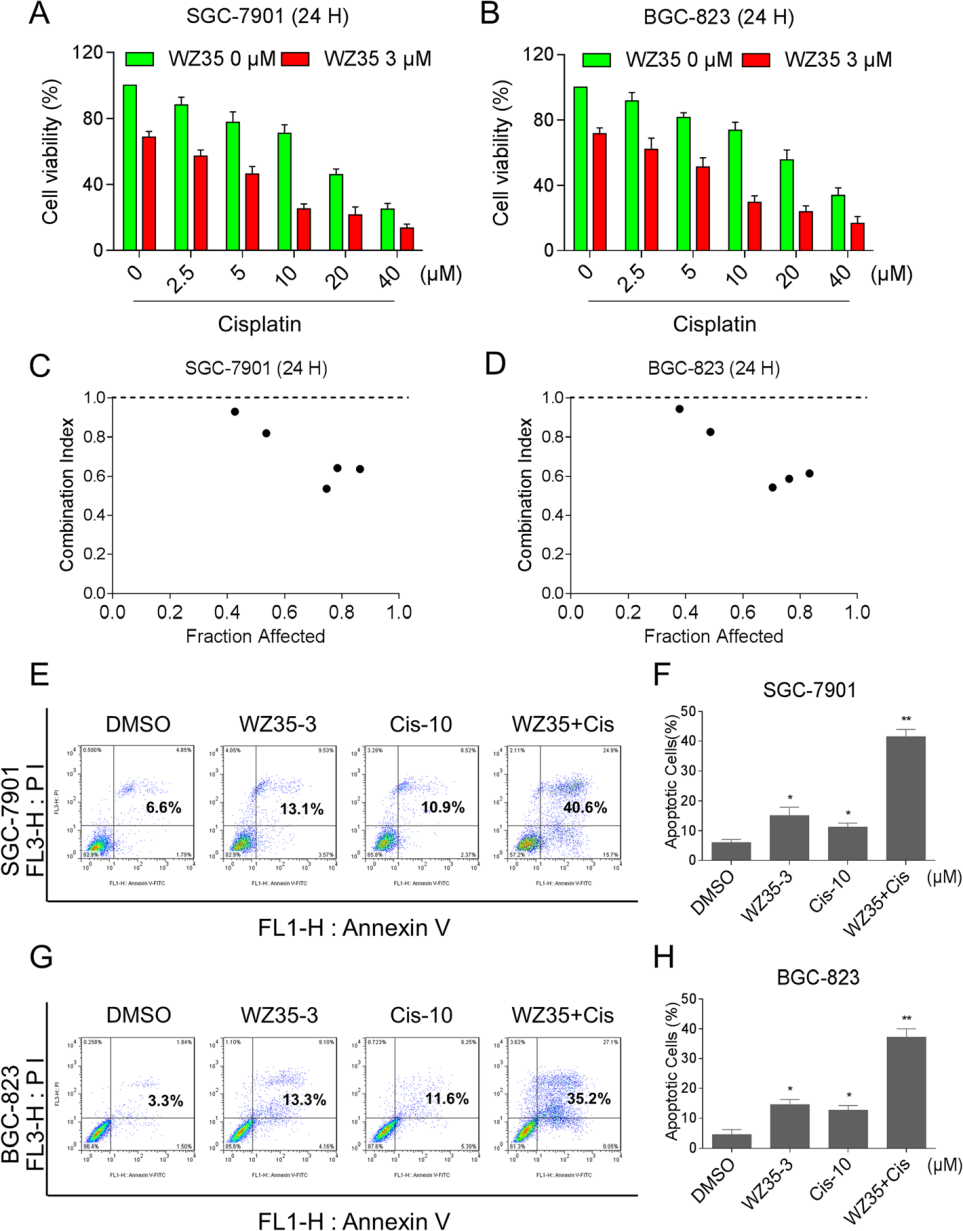
WZ35 synergistically increased the cytotoxicity of cisplatin in gastric cancer cells. (**a**-**b**) SGC-7901 or BGC-823 cells were treated with WZ35 or cisplatin alone or their combination at the indicated doses. At 24 h after treatment, the cell viability was determined by MTT assay. (**c**-**d**) The combination index (CI) values of WZ35 combined with cisplatin were calculated using the calcusyn software. (**e**-**h**) SGC-7901 or BGC-823 cells were treated with WZ35 or cisplatin alone or their combination at the indicated doses. At 24 h after treatment, the percentage of cell apoptosis was determined by Annexin-V/PI staining and flow cytometry, and the percentage of apoptotic cells in the treatment groups was calculated. (* *p* < 0.05, ** *p* < 0.01)

The cytotoxic effect of WZ35 was examined in human gastric cancer cells and normal cells. We found that WZ35 treatment preferentially suppressed the growth of gastric cancer cells in a dose-dependent manner (Additional file 1: Figure S1A-S1B). By contrast, WZ35 treatment has little effect on normal HL-7702 and NRK-52E cells (Additional file 1: Figure S1C-S1D). To determine whether WZ35 might synergize with cisplatin to kill gastric cancer cells, we examined the effect of WZ35 or cisplatin alone or their combination on cell viability in SGC-7901 and BGC-823 cells. The MTT assay showed that 3 μM WZ35 significantly increased the cytotoxicity of cisplatin in SGC-7901 and BGC-823 cells (Fig. 1a-b and Additional file 1: Figure S2A-S2B). Drug interaction of WZ35 and cisplatin was calculated by combination index values (Fig. 1c-d and Additional file 1: Figure S2C-S2D), which demonstrated that WZ35 in combination with cisplatin exhibited a synergistic effect in gastric cancer cells. Furthermore, compared with WZ35 or cisplatin treatment alone, the combined treatment dramatically increased the apoptotic cell death in both SGC-7901 and BGC-823 cells (Fig. 1e-h). These results suggest that WZ35 synergized the chemotherapeutic effect of cisplatin in gastric cancer.

### WZ35 and cisplatin cooperated to trigger ROS-dependent apoptosis in gastric cancer cells

**Fig. 2:**
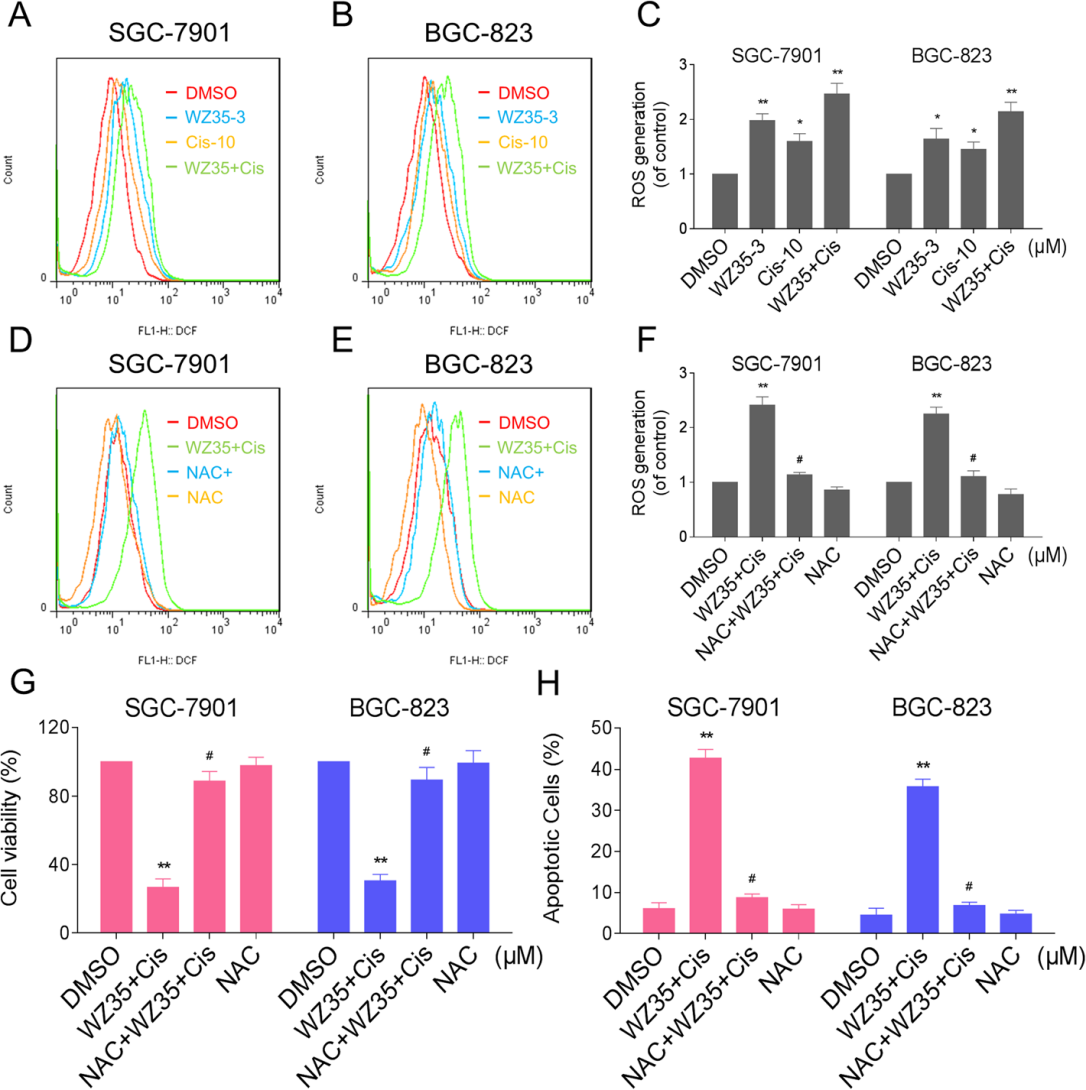
WZ35 and cisplatin cooperated to trigger ROS-dependent apoptosis in gastric cancer cells. (**a**-**c**) SGC-7901 or BGC-823 cells were treated with WZ35 or cisplatin alone or their combination at the indicated doses. At 2 h after treatment, intracellular ROS generation was determined by flow cytometry. (**d**-**f**) SGC-7901 or BGC-823 cells were pretreated with 5 mM NAC for 2 h before the combined treatment. Intracellular ROS generation was measured by flow cytometry. (**g**) SGC-7901 or BGC-823 cells were pretreated with 5 mM NAC for 2 h before exposure to WZ35 and cisplatin combination. At 24 h after treatment, the cell viability was determined by MTT assay. (**h**) SGC-7901 or BGC-823 cells were pretreated with 5 mM NAC for 2 h before exposure to WZ35 and cisplatin combination for 24 h. Percentage of cell apoptosis was determined by flow cytometry. The percentage of apoptotic cells in the treatment groups was calculated. Data represent similar results from three independent experiments. (* *p* < 0.05, ** *p* < 0.01)

The current study showed that WZ35 in combination with cisplatin exhibited a synergistic effects in SGC-7901 and BGC-823 cells. Hence, it is of great significance to investigate the synergistic mechanisms of WZ35 and cisplatin. In our previous study, we have demonstrated that WZ35 induces apoptosis by increasing the levels of intracellular ROS [13]. To demonstrate whether ROS was involved in the synergistic effect, we detected the intracellular ROS levels after WZ35 and cisplatin co-treatment. As shown in Fig. 2a-c, treatment of cells with WZ35 or cisplatin alone both induced ROS generation, but the combined treatment resulted in significant increases in ROS levels. To confirm whether ROS accumulation is a necessary event in the synergistic effect, the ROS scavenger NAC was used in our experiment. As shown in Fig. 2d-f, pretreatment with NAC markedly reversed the combined treatment-induced increase in ROS levels. The MTT assay revealed that scavenging of ROS markedly attenuated the combined treatment-induced cell growth inhibition in both SGC-7901 and BGC-823 cells (Fig. 2g and Additional file 1: Figure S3A-S3B). Moreover, it was found that pretreatment with NAC significantly abrogated the combined treatment-induced apoptosis in SGC-7901 and BGC-823 cells (Fig. 2h). These results revealed the vital role of ROS in the synergistic effect of WZ35 and cisplatin.

### WZ35 and cisplatin combination activated ROS-mediated p38 and JNK signaling pathways

**Fig. 3:**
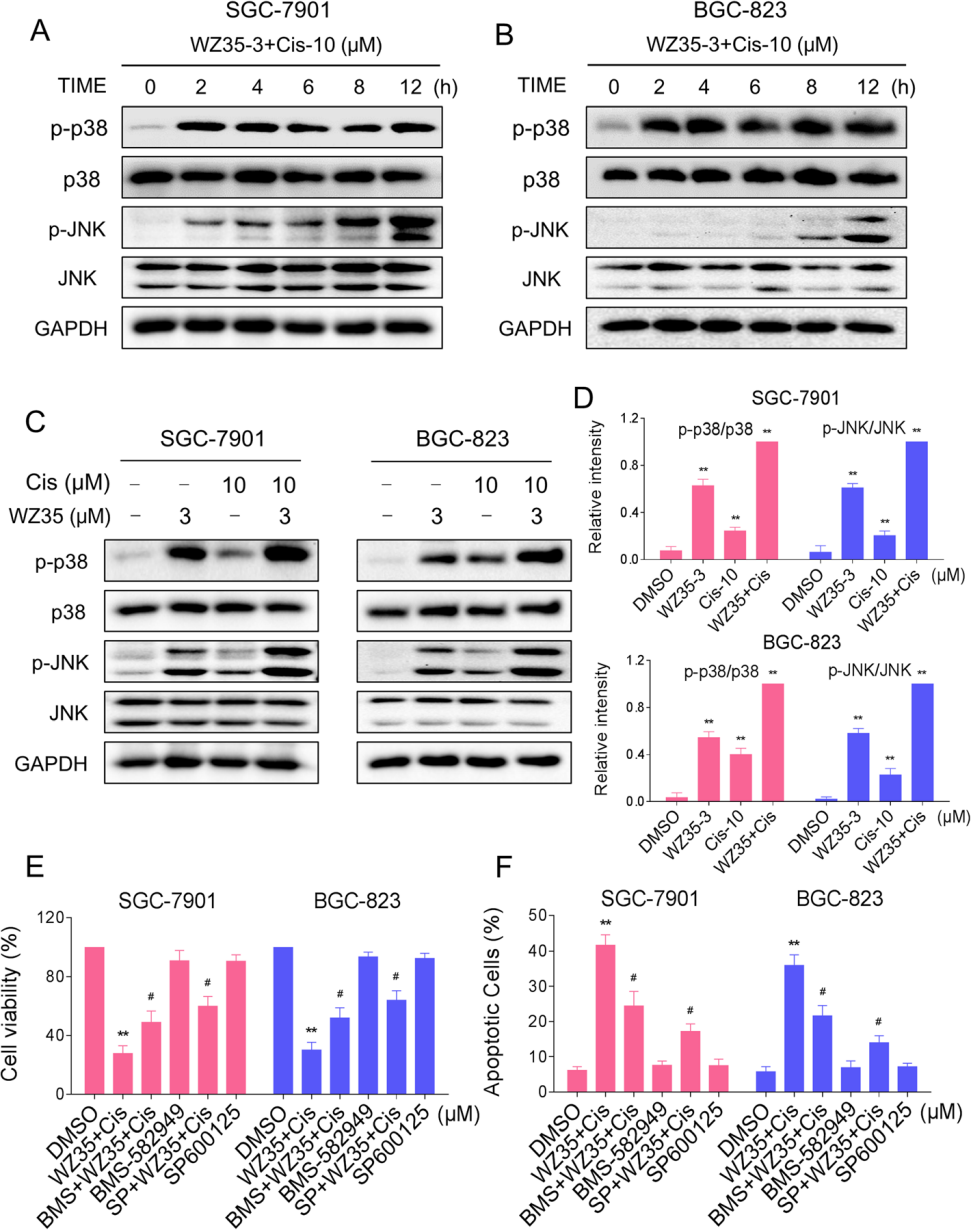
WZ35 and cisplatin combination activated p38 and JNK signaling pathways. (**a**-**b**) SGC-7901 or BGC-823 cells were treated with WZ35 and cisplatin combination for the indicated times, the protein levels of p-p38, p38, p-JNK and JNK were determined by western blot. GAPDH was used as the internal control. (**c**-**d**) SGC-7901 or BGC-823 cells were treated with WZ35 or cisplatin alone or their combination at the indicated doses. At 12 h after treatment, the protein levels of p-p38, p38, p-JNK and JNK were determined by western blot. GAPDH was used as the internal control. (**e**) SGC-7901 or BGC-823 cells were pretreated with BMS-582949 (10 μM) or SP600125 (20 μM) for 2 h before exposure to WZ35 and cisplatin combination for 24 h, the cell viability was determined by MTT assay. (**f**) SGC-7901 or BGC-823 cells were pretreated with BMS-582949 (10 μM) or SP600125 (20 μM) for 2 h before exposure to WZ35 and cisplatin combination for 24 h, percentage of cell apoptosis was determined by flow cytometry. The percentage of apoptotic cells in the treatment groups was calculated. Data represent similar results from three independent experiments. (* *p* < 0.05, ** *p* < 0.01)

In response to ROS, the oxidized Trx form is released and activates ASK1 to mediate apoptosis via the p38 and JNK signaling pathways [24, 25]. Therefore, we hypothesize that activation of p38 and JNK signaling pathways contributes to gastric cancer cells apoptosis induced by the combined treatment. As shown in Fig. 3a-b, the combined treatment could significantly activates p38 and JNK signaling pathways in both SGC-7901 and BGC-823 cells. Furthermore, compared with WZ35 or cisplatin treatment alone, the combined treatment resulted in more significant increases in the phosphorylation levels of p38 and JNK (Fig. 3c-d). To further validate the involvement of the p38 and JNK signaling pathways in WZ35 and cisplatin-mediated cell growth inhibition and apoptosis, the SGC-7901 and BGC-823 cells were co-treated with WZ35 and cisplatin after pretreatment with p38 inhibitor BMS-582949 or JNK inhibitor SP600125. As shown in Additional file 1: Figure S4A-S4B, we detected the inhibiting efficiencies of p-p38 and p-JNK by BMS-582949 and SP600125 respectively. We found that pretreatment with BMS-582949 or SP600125 markedly reversed the combined treatment-induced phosphorylation of p38 or JNK in SGC-7901 cells. Moreover, the results in Fig. 3e-f showed that BMS-582949 or SP600125 can partially attenuate combined treatment-induced cell growth inhibition and apoptosis, suggesting that the activation of p38 and JNK signaling pathways is essential for the lethality of combined treatment.

**Fig. 4:**
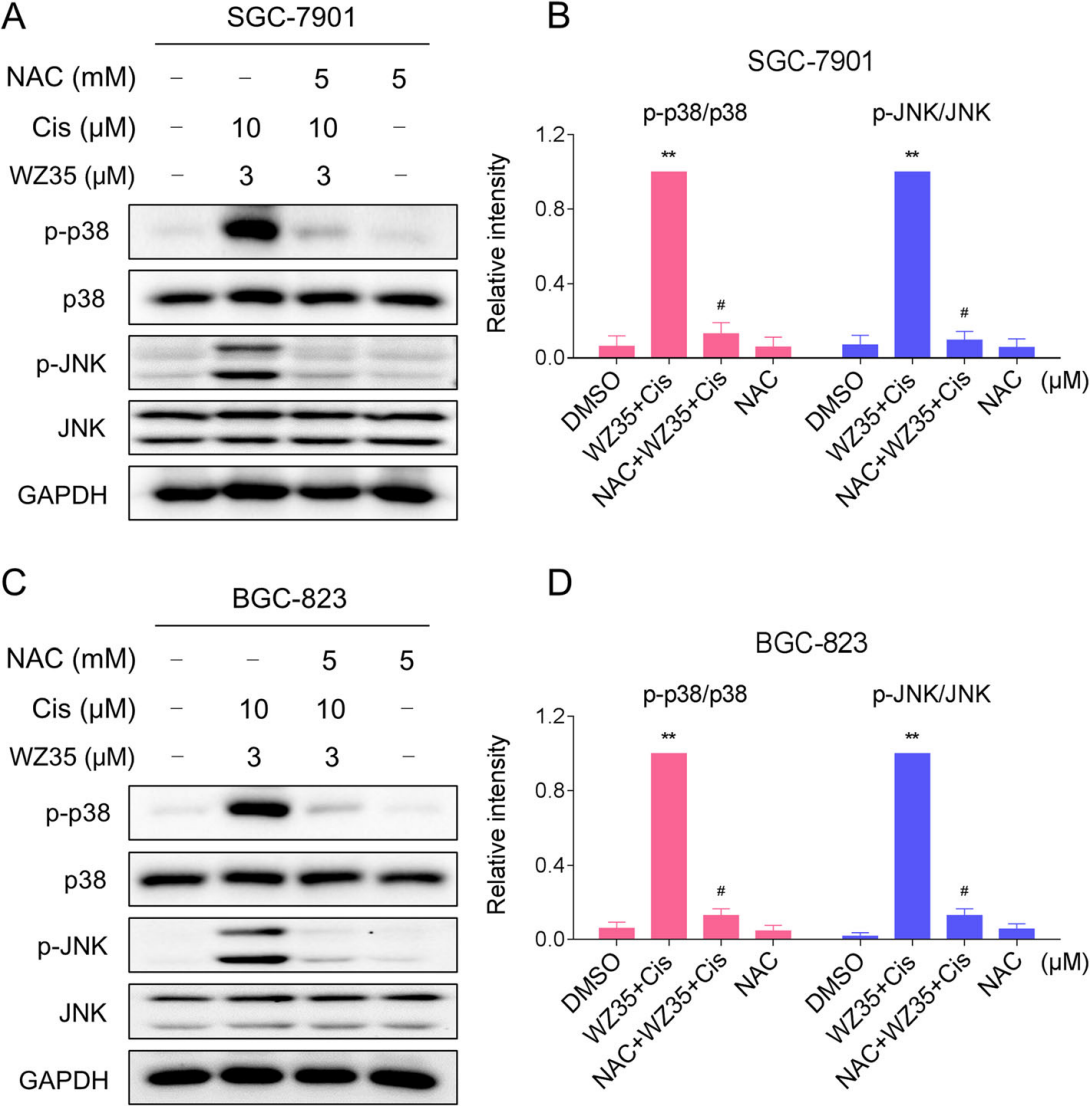
The activation of p38 and JNK signaling pathways is dependent on intracellular ROS generation. (**a**-**b**) SGC-7901 cells were pretreated with NAC (5 mM) for 2 h before exposure to WZ35 and cisplatin combination. At 12 h after treatment, the protein levels of p-p38, p38, p-JNK and JNK were determined by western blot. GAPDH was used as the internal control. (**c**-**d**) BGC-823 cells were pretreated with NAC (5 mM) for 2 h before exposure to WZ35 and cisplatin combination. At 12 h after treatment, the protein levels of p-p38, p38, p-JNK and JNK were determined by western blot. GAPDH was used as the internal control. Data represent similar results from three independent experiments. (* *p* < 0.05, ** *p* < 0.01)

We next tested the connection between ROS accumulation and activation of p38 and JNK signaling pathways. We observed that pretreatment with NAC markedly reversed the combined treatment-induced phosphorylation of p38 and JNK in both SGC-7901 and BGC-823 cells (Fig. 4a-d). Taken together, these results suggest that the activation of p38 and JNK signaling pathways is due to accumulation of intracellular ROS in gastric cancer cells.

### WZ35 and cisplatin combination inhibited TrxR1 activity in gastric cancer cells

**Fig. 5:**
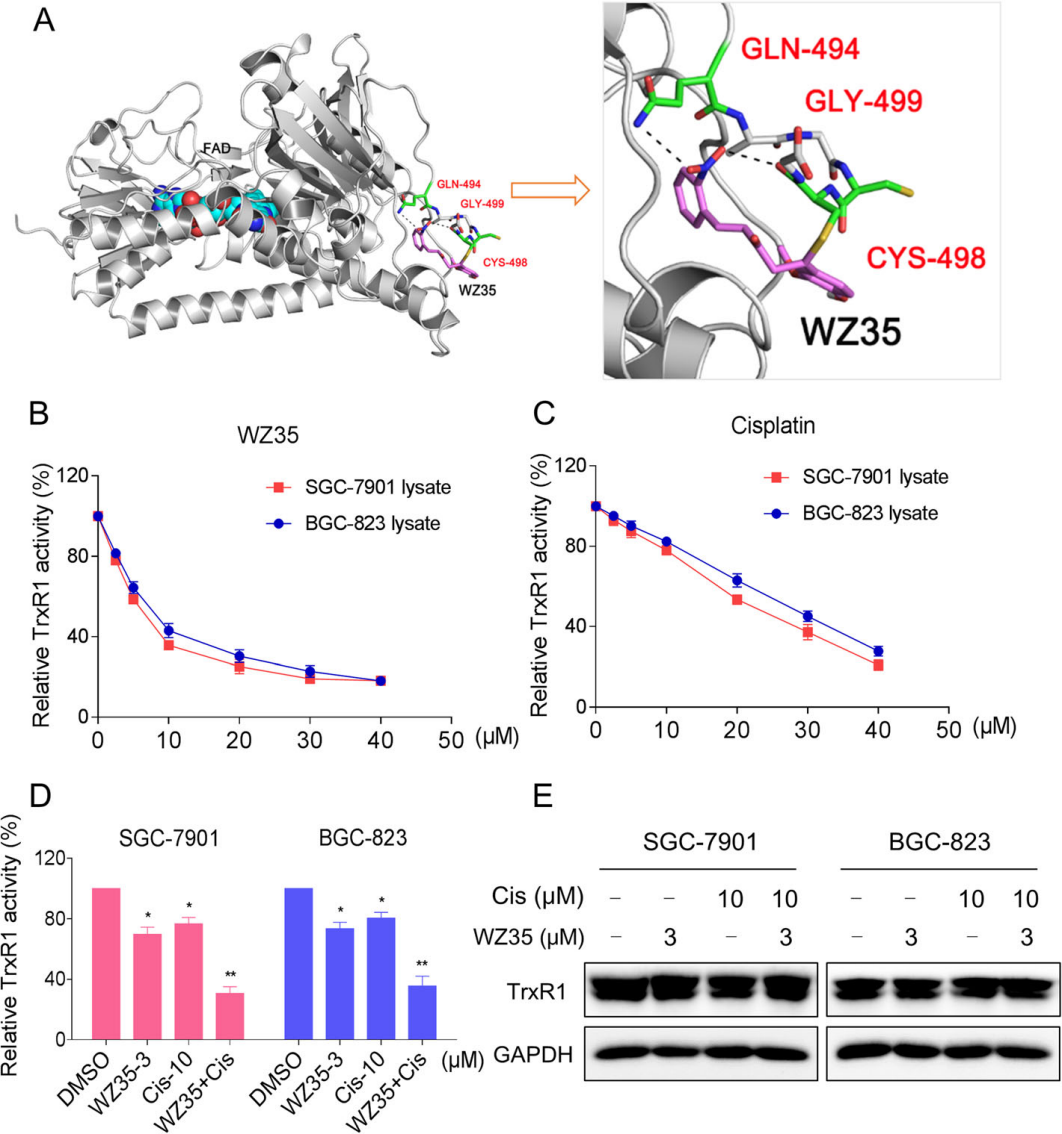
WZ35 and cisplatin combination inhibited TrxR1 activity in gastric cancer cells. (**a**) Molecular docking of WZ35 with TrxR1 protein was carried out with the docking software. (**b**-**c**) TrxR1 activity was measured in SGC-7901 or BGC-823 cells after treated with WZ35 or cisplatin. (**d**) SGC-7901 or BGC-823 cells were treated with WZ35 or cisplatin alone or their combination at the indicated doses. At 2 h after treatment, TrxR1 activity was measured by the endpoint insulin reduction assay. (**e**) SGC-7901 or BGC-823 cells were treated with WZ35 or cisplatin alone or their combination at the indicated doses. At 12 h after treatment, the TrxR1 expression was determined by western blot. Data represent similar results from three independent experiments. (* *p* < 0.05, ** *p* < 0.01)

TrxR1, which catalyzes the NADPH-dependent reduction of thioredoxin, is an important regulator of redox balance in cells. Accumulating evidence suggests that intracellular ROS might be increased when the TrxR1 activity was chemically inhibited [23, 26, 27]. Therefore, we first performed a molecular simulation of WZ35-TrxR1 complex using docking software. As shown in Fig. 5a, the michael acceptor of WZ35 form a covalent bond with the residue Cys-498 of the C-terminal active site redox center of TrxR1. Additionally, WZ35 interacts with the residues GLN-494 and GLY-499 through the formation of hydrogen bonds. Further, we tested the inhibitory effects of WZ35 on TrxR1 enzyme activity by using the endpoint insulin reduction assay, we found that TrxR1 activity in cell lysates was decreased with increasing WZ35 concentration (Fig. 5b). Interestingly, TrxR1 activity was also inhibited by cisplatin in a dose-dependent manner, and the combined treatment exerted a stronger inhibitory effect on TrxR1 activity than either WZ35 or cisplatin alone in both SGC-7901 and BGC-823 cells (Fig. 5c-d). The western blot analysis revealed that the expression of TrxR1 had no significant change after treatment with WZ35 or cisplatin alone or their combination (Fig. 5e). These results indicate that the effects of WZ35 and cisplatin to induce ROS is linked to their ability to inhibit TrxR1 activity.

### WZ35 and cisplatin combination inhibited SGC-7901 xenograft tumor growth in vivo*,* accompanied by decreased TrxR1 activity

**Fig. 6:**
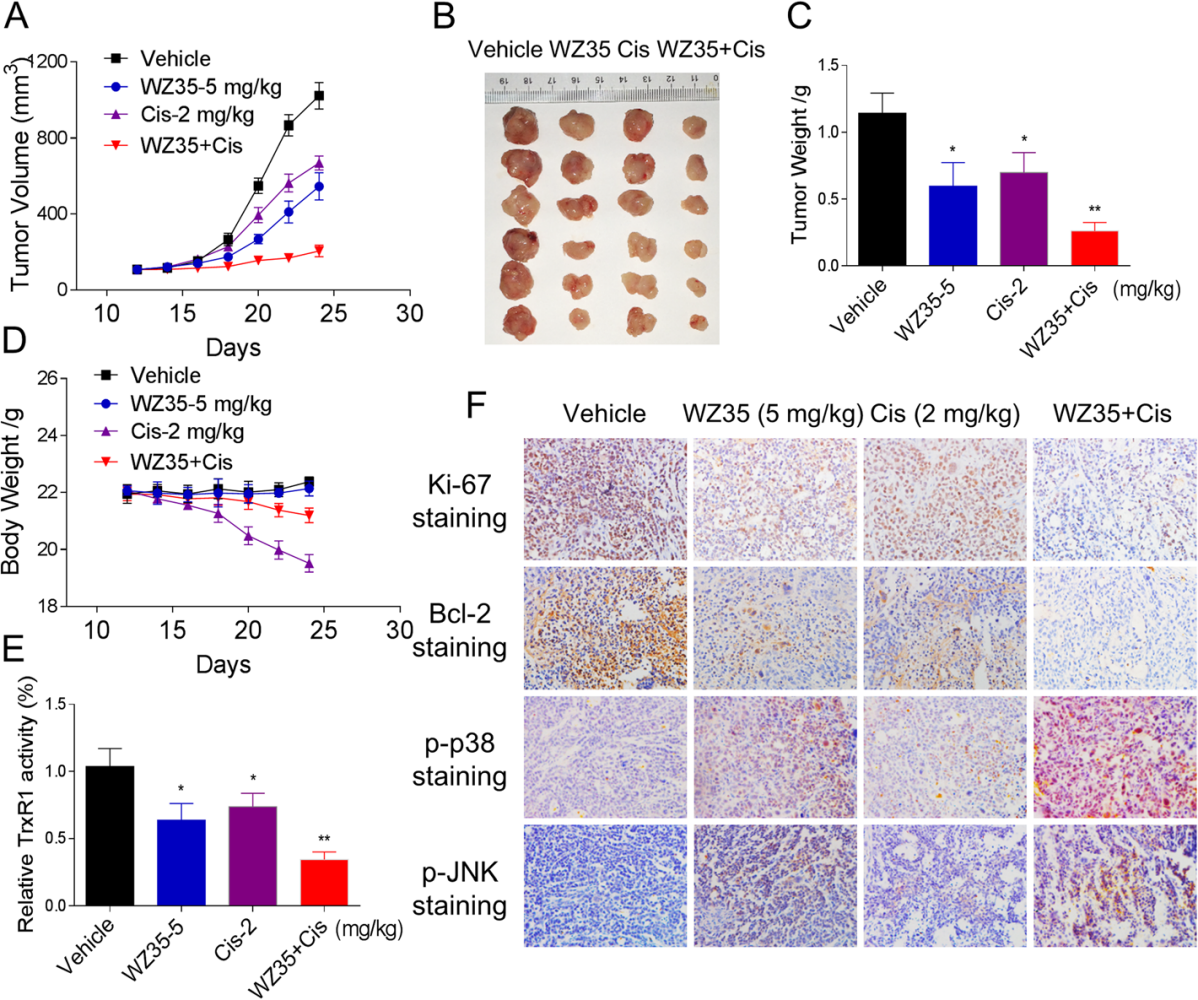
WZ35 and cisplatin combination inhibited SGC-7901 xenograft tumor growth in vivo*,* accompanied by decreased TrxR1 activity. (**a**-**b**) WZ35 and cisplatin combined treatment significantly inhibited tumor volume and tumor weight (**c**) of SGC-7901 human gastric cancer xenografts in nude mice. (**d**) The body weight of nude mice during the experiment. (**e**) The tumor tissues were lysed and protein was used to determine the TrxR1 activity by using the endpoint insulin reduction assay. (**f**) The expression levels of Ki-67, Bcl-2, p-p38 and p-JNK proteins in tumor tissues were analyzed by immunohistochemical analysis

To evaluate the in vivo effect of the combined treatment, we used a subcutaneous xenograft model of SGC-7901 cells in immunodeficient mice. After 13 days treatment, we found that 5 mg/kg WZ35 and 2 mg/kg cisplatin showed effective inhibition on the growth of SGC-7901 xenograft (Fig. 6a-c). However, the combined treatment exhibited stronger inhibitory effects on tumor volume and weight (Fig. 6a-c). Remarkably, the administration of cisplatin (2 mg/kg) resulted in a significant weight loss, whereas the combined treatment was well tolerated, suggesting that WZ35 can attenuate the side effects of cisplatin (Fig. 6d). We further validated this result by using ICR mice. We found that WZ35 treatment remarkably attenuated the decrease of body weight and spleen weight evoked by cisplatin treatment, and decreased the gastric residual volume and serum BUN levels induced by cisplatin (Additional file 1: Figure S5A-S5F). Mechanistically, TrxR1 activity in tumor tissues was measured by endpoint insulin reduction assay, and the result indicated that combined treatment significantly decreased the activity of TrxR1 (Fig. 6e). Moreover, the immunohistochemical staining assay was used to determine the expression of Ki-67, Bcl-2, p-p38 and p-JNK. The expression levels of Ki-67 and Bcl-2 were significantly decreased, whereas p-p38 and p-JNK were increased by the combined treatment (Fig. 6f). Taken together, these results indicate that WZ35 could synergize the effect of cisplatin to inhibit tumor growth in vivo by targeting TrxR1, which was in accordance with the mechanisms in vitro.

## Discussion

**Fig. 7:**
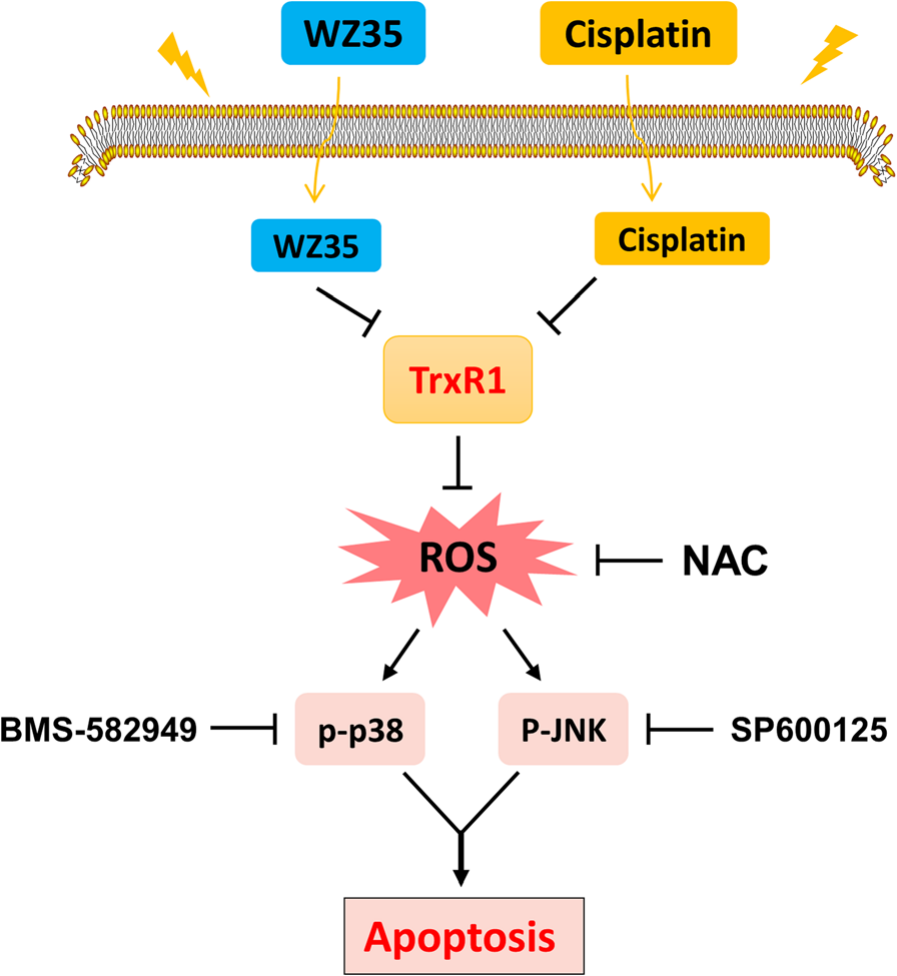
Schematic illustration of the main findings of the present study

In this study, we investigated the response of human gastric cancer cells to the combined treatment of WZ35 and cisplatin. Our results showed that the enhanced inhibitory effect of WZ35 and cisplatin on tumor cell growth was mediated through inhibiting TrxR1 activity. By inhibiting TrxR1 activity, WZ35 combined with cisplatin markedly induced the production of ROS, activated p38 and JNK signaling pathways, and eventually induced apoptosis of gastric cancer cells (Fig. 7). In vivo, WZ35 combined with cisplatin exhibited a synergistic inhibitory effect on tumor growth, and effectively reduced the activity of TrxR1 in tumor tissues, which was consistent with in vitro study. Remarkably, WZ35 attenuated the body weight loss evoked by cisplatin treatment. It would be important to clarify the underlying mechanisms in the future study.

Our previous studies have shown that WZ35 was able to selectively induce apoptosis in cancer cells by increasing intracellular ROS levels [13, 28]. Therefore, we were interested in whether ROS was involved in the synergistic effect of WZ35 and cisplatin. In our study, we found that WZ35 and cisplatin combined treatment resulted in significant increases in ROS levels, and pretreatment with NAC fully reversed the combined treatment-induced ROS generation and apoptosis, suggesting that ROS play a critical role in the synergistic effect of WZ35 and cisplatin. By inducing ROS generation and oxidative stress, the combined treatment concomitantly activated p38 and JNK signaling pathways, as indicated by phosphorylation of both p38 and JNK. Moreover, we observed that pretreatment with NAC markedly reversed the combined treatment-induced phosphorylation of p38 and JNK in both SGC-7901 and BGC-823 cells, suggesting that the activation of p38 and JNK signaling pathways is due to accumulation of intracellular ROS. These results indicate that WZ35 might be a novel agent to augment ROS production and sensitize cancer cells to chemotherapeutic drugs.

The combination of WZ35 and cisplatin resulted in significant increases in ROS levels. However, the molecular mechanism underlying combined treatment-induced ROS production remains unknown. TrxR1 is a selenoprotein that functions to reduce the oxidoreductase thioredoxin (Trx) in a NADPH dependent, and plays an important role in regulating the redox balance in cells. Accumulating evidence suggests that intracellular ROS might be increased when the TrxR1 activity was chemically inhibited [23, 26, 27]. TrxR1 has emerged as a promising therapeutic target in cancer chemotherapy, because TrxR1 have been shown to be overexpressed in a variety of cancer cells and human tumors, and associated with increased tumor growth and poor patient prognosis [15, 16, 29]. Therefore, the past years have witnessed an increasing interest in developing novel TrxR1 inhibitors as potential antitumor agents [30, 31]. Using the the endpoint insulin reduction assay to quantify inhibition of TrxR1 activity, we found that TrxR1 activity in cell lysates was decreased with increasing WZ35 concentration. Interestingly, TrxR1 activity was also inhibited by cisplatin in a dose-dependent manner, and the combined treatment exerted a stronger inhibitory effect on TrxR1 activity than either WZ35 or cisplatin alone in both SGC-7901 and BGC-823 cells. Taken together, our results provide a satisfying mechanistic explanation for previous observations: namely that WZ35 and cisplatin can inhibit TrxR1 activity, inducing ROS generation and in some cells the induction of cell death.

## Conclusions

In conclusion, we found that WZ35 synergized the anti-tumor effect of cisplatin by inhibiting TrxR1 activity, and demonstrated that the combined treatment induced apoptotic cell death through ROS-mediated p38 and JNK signaling pathways. These findings provide new insights into the molecular mechanisms by which WZ35 synergized with cisplatin, and suggest that such a combinatorial treatment might potentially become a more effective way in gastric cancer therapy. Further investigations about the clinical significance of this combination therapy are needed.

## Additional file

Additional file 1: **Figure S1** WZ35 selectively inhibits the growth of gastric cancer cells. **Figure S2** WZ35 synergistically increased the cytotoxicity of cisplatin in gastric cancer cells. **Figure S3** Pretreatment with GSH markedly attenuated the combined treatment-induced cell growth inhibition in gastric cancer cells. **Figure S4** The inhibiting efficiencies of p-p38 and p-JNK by BMS-582949 and SP600125 respectively. **Figure S5** WZ35 reduced the toxicity of cisplatin in vivo. (DOCX 681 kb)

## Abbreviations

CI: combination index
DAB: 3,3′-diaminobenzidine
NAC: N-acetyl-L-cysteine
ROS: reactive oxygen species
Sec: selenocysteine
TrxR1: thioredoxin reductase 1
