# Supplementary material for: Curcuminoid WZ35 synergize with cisplatin by inducing ROS production and inhibiting TrxR1 activity in gastric cancer cells
Source: J Exp Clin Cancer Res. 2019 May 21;38:207. doi: 10.1186/s13046-019-1215-y (PMC6528260; doi:10.1186/s13046-019-1215-y)
Supplement: Supplementary file 1 — Figure S1 WZ35 selectively inhibits the growth of gastric cancer cells. Figure S2 WZ35 synergistically increased the cytotoxicity of cisplatin in gastric cancer cells. Figure S3 Pretreatment with GSH markedly attenuated the combined treatment-induced cell growth inhibition in gastric cancer cells. Figure S4 The inhibiting efficiencies of p-p38 and p-JNK by BMS-582949 and SP600125 respectively. Figure S5 WZ35 reduced the toxicity of cisplatin in vivo. (DOCX 681 kb) [file 13046_2019_1215_MOESM1_ESM.docx]

***Supporting Information***

**Curcuminoid WZ35 synergize with cisplatin by inducing ROS production and inhibiting** [**TrxR1**](https://www.ncbi.nlm.nih.gov/pubmed/26919110)**activity in gastric cancer cells**

Wei He^1#^, Yiqun Xia^2#^, Peihai Cao^1#^, Lin Hong^1^, Tingting Zhang^1^, Xin Shen^1^, Peisen Zheng^1^, Huanpei Shen^1^, Guang Liang^1^*, Peng Zou^1^*

^1^ Chemical Biology Research Center, School of Pharmaceutical Sciences, Wenzhou Medical University, Wenzhou, Zhejiang 325035, China

^2^ Department of Digestive Diseases, The First Affiliated Hospital of Wenzhou Medical University, Wenzhou, Zhejiang 325035, China

* **Corresponding author:** Peng Zou, Ph.D

Address: Chemical Biology Research Center, School of Pharmaceutical Sciences, Wenzhou Medical University, Wenzhou 325035, China

Tel: +86-577-86699892; Fax: +86-577-86689982

E-mail: zoupeng123@163.com

* Co-corresponding author: Guang Liang, Ph.D

Address: Chemical Biology Research Center, School of Pharmaceutical Sciences, Wenzhou Medical University, Wenzhou 325035, China

Tel: +86-577-86699892; Fax: +86-577-86689982

E-mail: wzmuliangguang@163.com

# These authors contributed equally to this work

**Supplementary Figures and Legends**

**
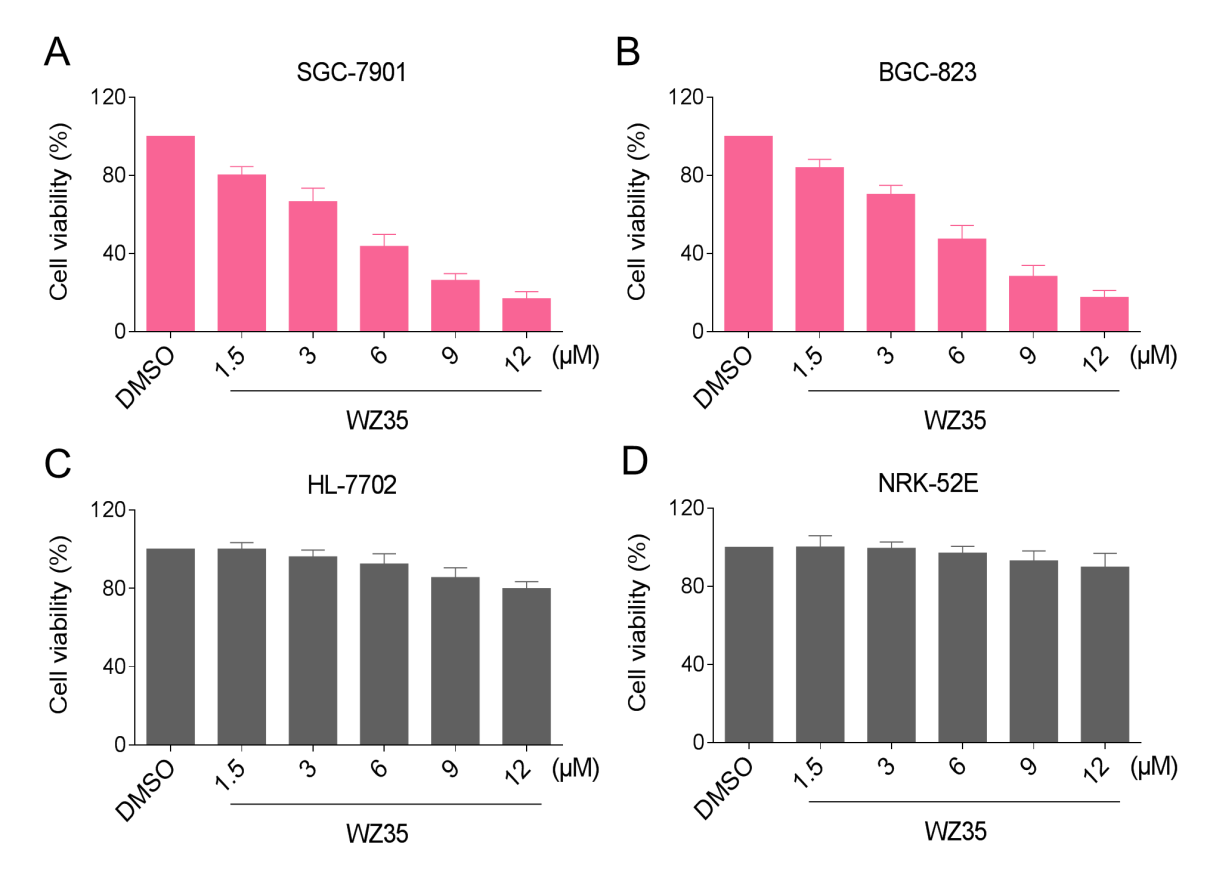
**

**Figure S1. WZ35 selectively inhibits the growth of gastric cancer cells.** (A-B) SGC-7901 or BGC-823 cells were treated with WZ35 at the indicated doses. At 24 h after treatment, the cell viability was determined by MTT assay. (C-D) HL-7702 or NRK-52E cells were treated with WZ35 at the indicated doses. At 24 h after treatment, the cell viability was determined by MTT assay.

**
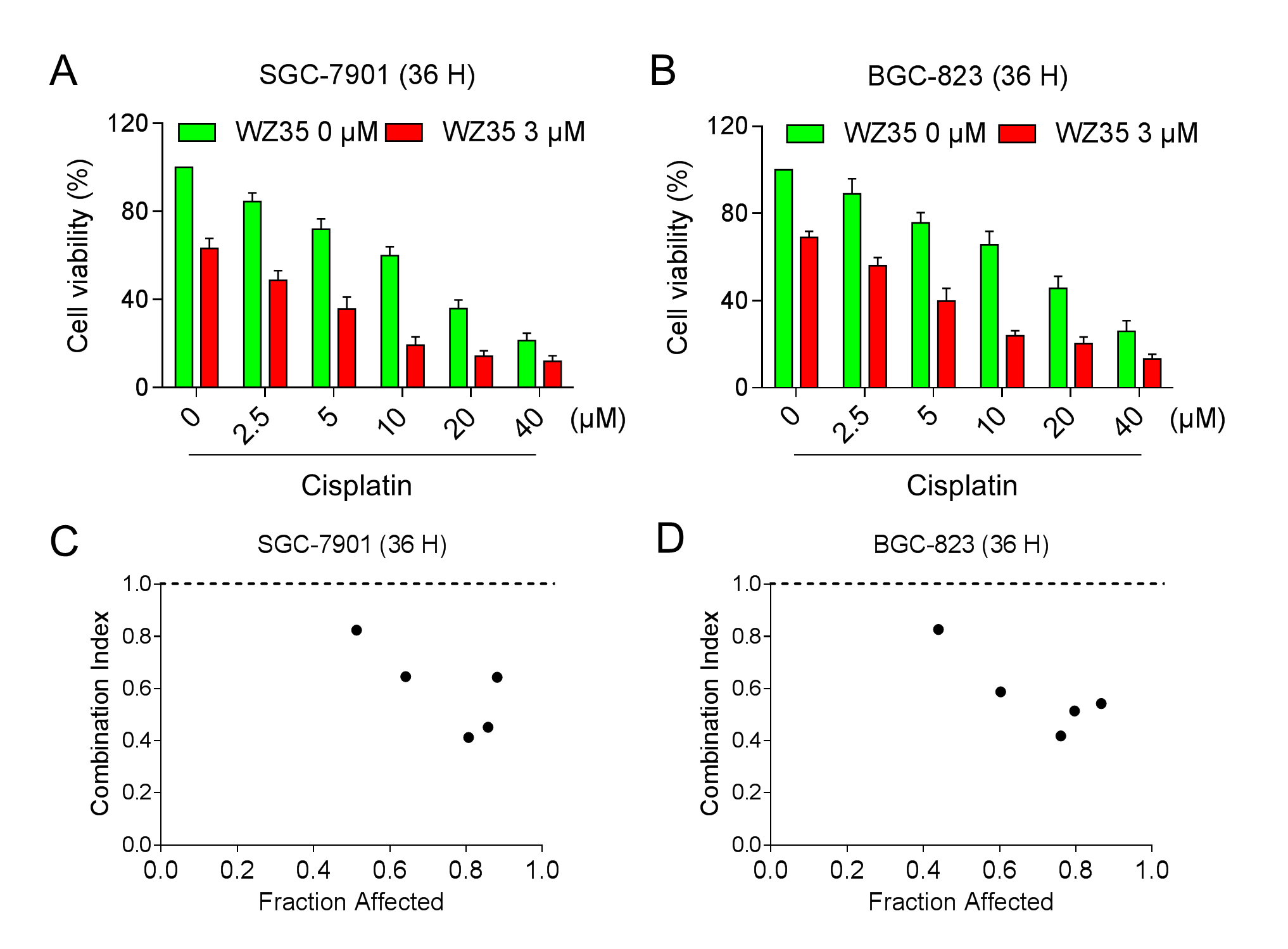
**

**Figure S2. WZ35 synergistically increased the cytotoxicity of cisplatin in gastric cancer cells.** (A-B) SGC-7901 or BGC-823 cells were treated with WZ35 or cisplatin alone or their combination at the indicated doses. At 36 h after treatment, the cell viability was determined by MTT assay. (C-D) The combination index (CI) values of WZ35 combined with cisplatin were calculated using the calcusyn software.

**
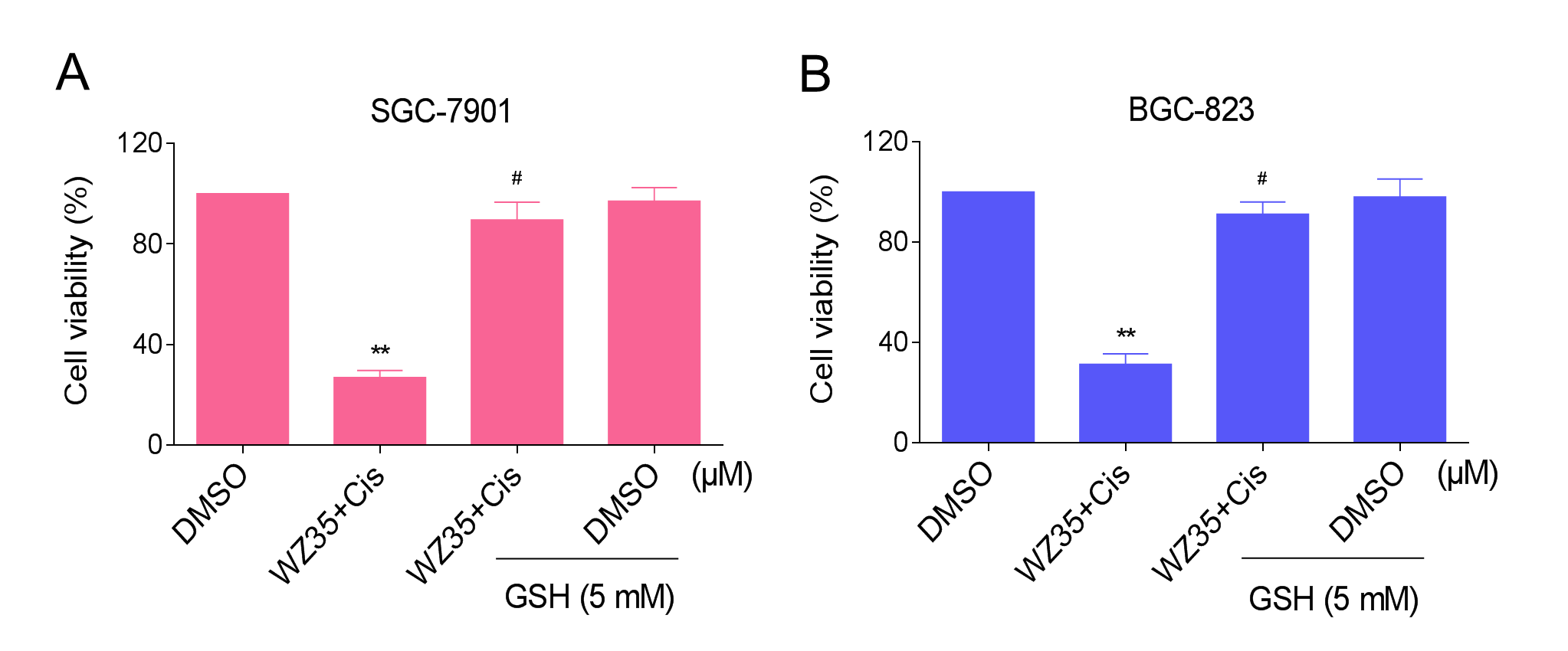
**

**Figure S3. Pretreatment with GSH markedly attenuated the combined treatment-induced cell growth inhibition in gastric cancer cells**. (A-B) SGC-7901 or BGC-823 cells were pretreated with 5 mM GSH for 2 h before exposure to WZ35 and cisplatin combination. At 24 h after treatment, the cell viability was determined by MTT assay.


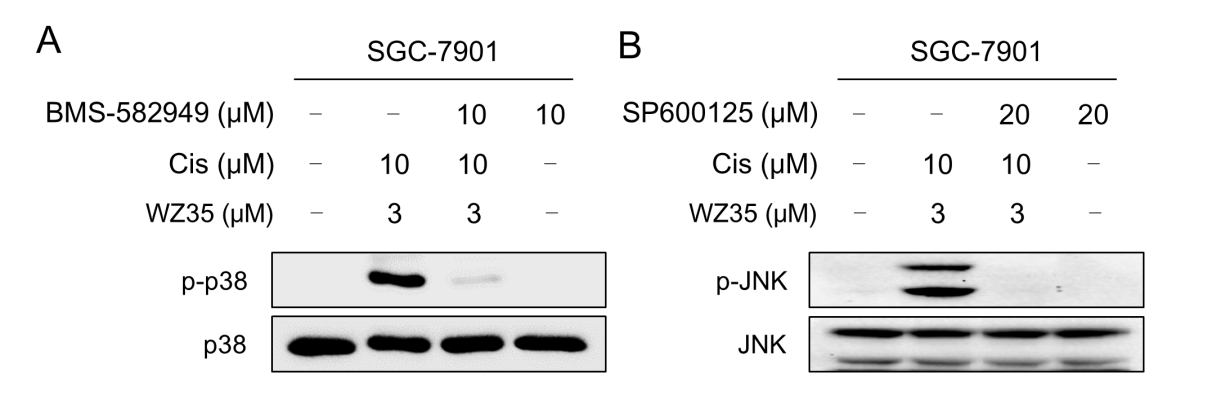


**Figure S4. The inhibiting efficiencies of p-p38 and p-JNK by BMS-582949 and SP600125 respectively.** (A-B) SGC-7901 cells were pretreated with BMS-582949 (10 μM) or SP600125 (20 μM) for 2 h before exposure to WZ35 and cisplatin combination. At 12 h after treatment, the protein levels of p-p38, p38, p-JNK and JNK were determined by western blot.


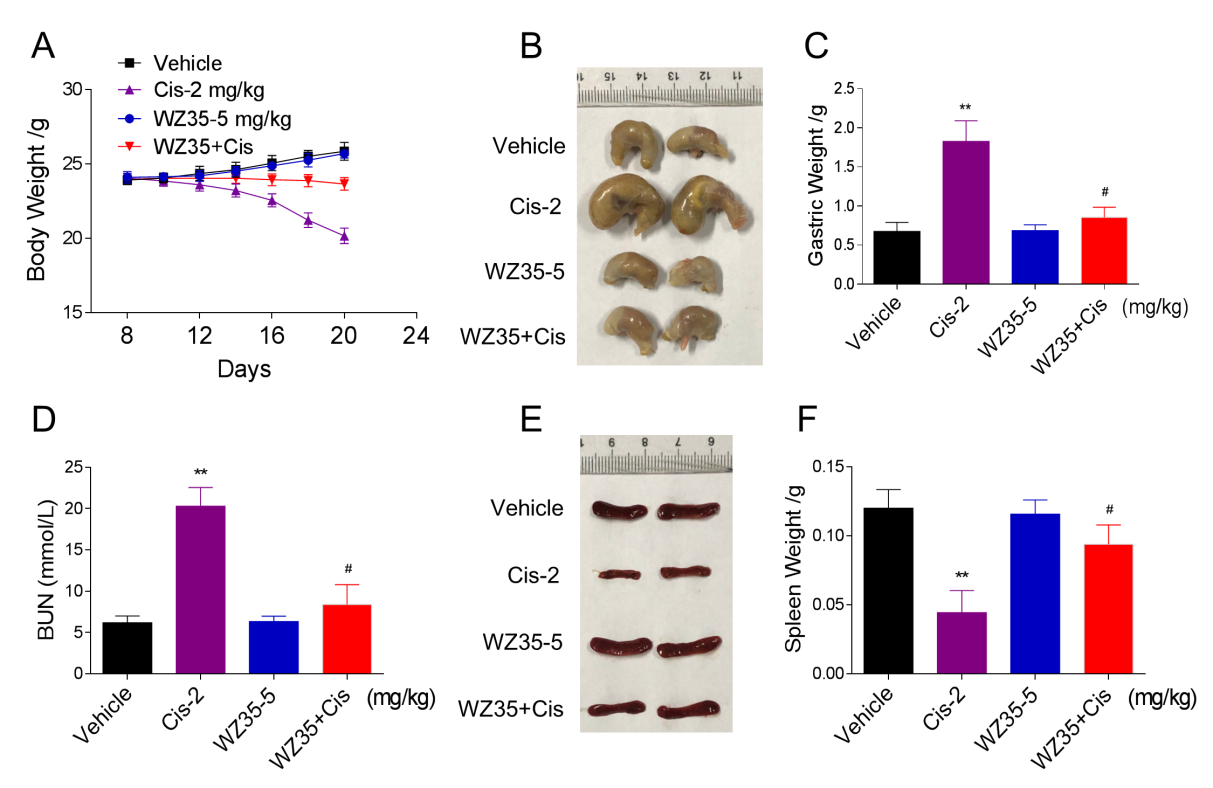


**Figure S5. WZ35 reduced the toxicity of cisplatin in *vivo*.** (A) The body weight of ICR mice during the experiment. (B-C) The gastric weight of mice after treatment. (D) The levels of serum blood urea nitrogen (BUN). (E-F) The spleen weight of mice after treatment.
